# A delayed presentation of traumatic right hemidiaphragm injury repaired via a laparoscopic approach: A case report

**DOI:** 10.1016/j.amsu.2020.03.002

**Published:** 2020-03-25

**Authors:** Bee S. Ong, Paul T. Heitmann, Jon Shenfine

**Affiliations:** Oesophagogastric Unit, Flinders Medical Centre, Adelaide, Australia

**Keywords:** Traumatic diaphragm injury, Delayed presentation, Laparoscopic approach, Mesh repair, Case report

## Abstract

**Introduction:**

Diaphragmatic injury is a rare clinical entity which presents a diagnostic and therapeutic challenge. It is three times more common following blunt trauma than penetrating trauma and results in larger tears. A high index of suspicion is required to diagnose diaphragmatic injury. A missed diagnosis following acute injury can later result in life-threatening complications.

**Case presentation:**

We describe the successful management of a right hemidiaphragmatic injury presenting two weeks following blunt thoracoabdominal trauma using a laparoscopic mesh repair.

**Discussion:**

Diaphragmatic injury is rare, with right-sided injuries less common due to the buffering effect of the liver. The diagnosis is made within 24 hours of injury in 75% of cases (Haranal and et al., 2018) [1]. In our patient, symptoms of a right-sided diaphragmatic injury manifested two weeks following a motor vehicle collision. A CT scan of the chest and abdomen confirmed the diagnosis. According to DeBlasio, intermittent symptoms of visceral herniation or incorrect x-ray interpretation are the main reasons for a delayed diagnosis (DeBlasio et al., 1994) [2]. Contrary to common practice where thoracotomy is the preferred method for repair in the absence of associated abdominal injuries, we demonstrated that a right-sided diaphragmatic injury can be successfully managed with a laparoscopic mesh repair.

**Conclusion:**

Traumatic diaphragmatic injury remains a challenge to emergency physicians and trauma surgeons. Clinicians should be aware of the differing clinical presentations, investigations, and management. Surgical repair can be achieved via laparoscopy, thoracoscopy, laparotomy, and/or thoracotomy. In the case of an isolated right-sided diaphragmatic injury, laparoscopic mesh repair should be considered.

## Highlights

• Diaphragmatic injury is uncommon and presents a diagnostic and therapeutic challenge.
• Injury can be identified in acute trauma management, but often the identification of diaphragmatic injury is delayed and manifests as atypical respiratory and/or abdominal symptoms.
• Computed tomography (CT) of the Chest and Abdomen remains the diagnostic modality of choice.
• Surgical repair of the defect can be achieved via laparoscopy, thoracoscopy, laparotomy, and/or thoracotomy.
• Laparoscopic repair of delayed diaphragmatic defect with mesh is achievable and avoids the morbidity associated with open surgery.

## 1 Introduction

Diaphragmatic injury is rare with an incidence of 0.8–5.0% following trauma [3]. Blunt trauma is three times more likely than penetrating trauma to cause diaphragmatic injury and often results in larger tears [4,5]. The diagnosis is made within 24 hours of injury in 75% of cases [1]. Missed injuries of the right hemidiaphragm are more common, which can remain unrecognised for weeks or even years [6]. According to DeBlasio, intermittent symptoms of visceral herniation or incorrect x-ray interpretation are the main reasons for a delayed diagnosis [2] However, with improved resolution and sensitivity of imaging, the reported incidence of right-sided diaphragmatic injury has increased. Surgical repair of the diaphragmatic defect is necessary to prevent complications from herniation of intra-abdominal contents into the thorax. We report a delayed presentation of a right diaphragmatic injury successfully repaired using a laparoscopic mesh repair. This case is reported in accordance with the SCARE criteria [7].

## 2 Case presentation

A 67-year-old man presented with orthopnoea two weeks after a high-speed, head-on motor vehicle collision. At the time of injury, he was wearing a seatbelt, self-extricated at the scene, and was assessed in a regional hospital. The patient's past medical history included hypertension and aortic and iliofemoral graft placement for peripheral vascular disease. His only regular medication was Olmesartan. Clinical assessment and computed tomographic (CT) imaging of the chest, abdomen, and pelvis identified an undisplaced fracture of the sternal head. The diaphragmatic injury was not initially identified. The patient was managed with analgesia and discharged home.

**Fig. 1:**
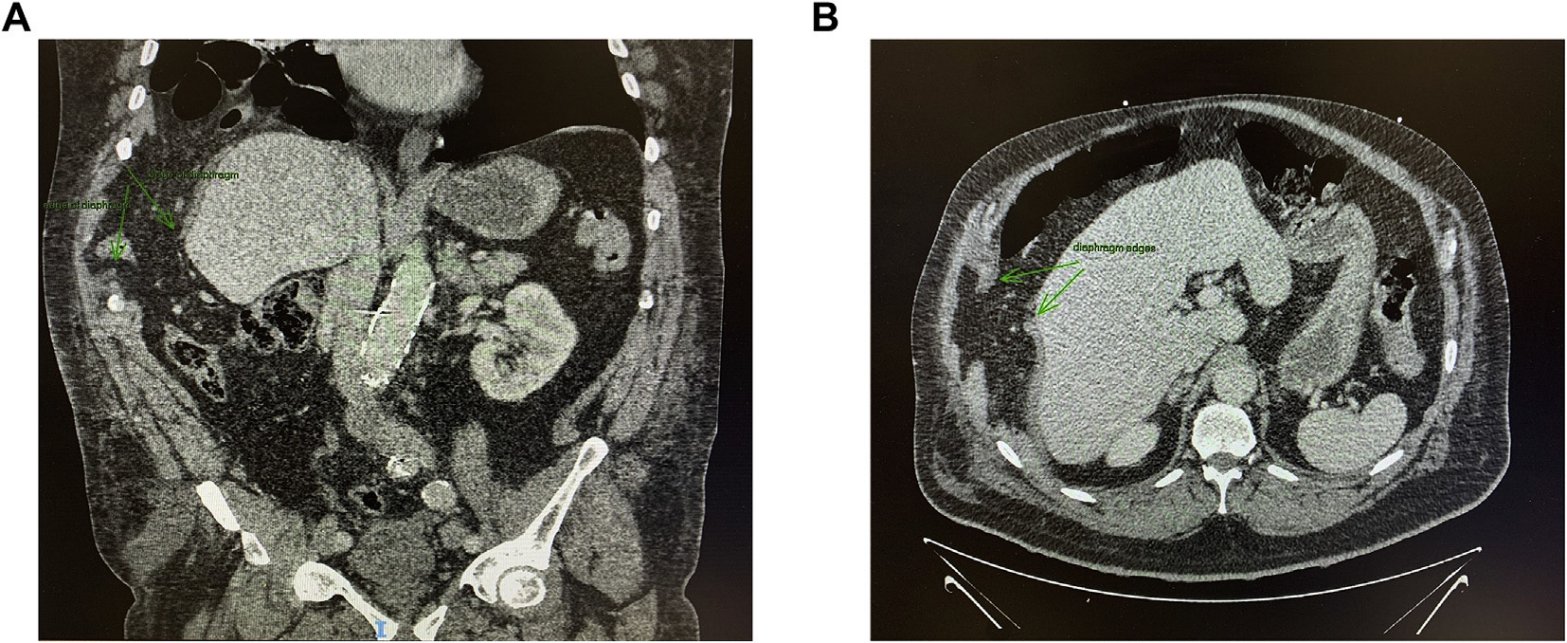
(a) (coronal view), (b) (axial view): CT scan showing diaphragmatic defect with herniation of bowel into right hemithorax.

The patient re-presented via the emergency department two weeks later with progressively worsening dyspnoea, orthopnoea, and right upper abdominal pain. On examination, he was dyspnoeic, requiring 4L/min of oxygen to maintain pulse oximetry of 95%. There was bruising visible on his right chest wall, subcostal region, and lower abdomen (“seatbelt sign”). The patient reported tenderness on palpation of his right subcostal region and right upper quadrant, however his abdomen was soft with no clinical signs of peritonism. Breath sounds were reduced at the right lung base. Blood investigations were unremarkable. Repeat x-ray and CT imaging revealed elevation of the right hemidiaphragm and herniation of bowel into the right hemithorax via a right hemidiaphragm defect, measuring 57mm in maximal width (Fig. 1a and b).

**Fig. 2:**
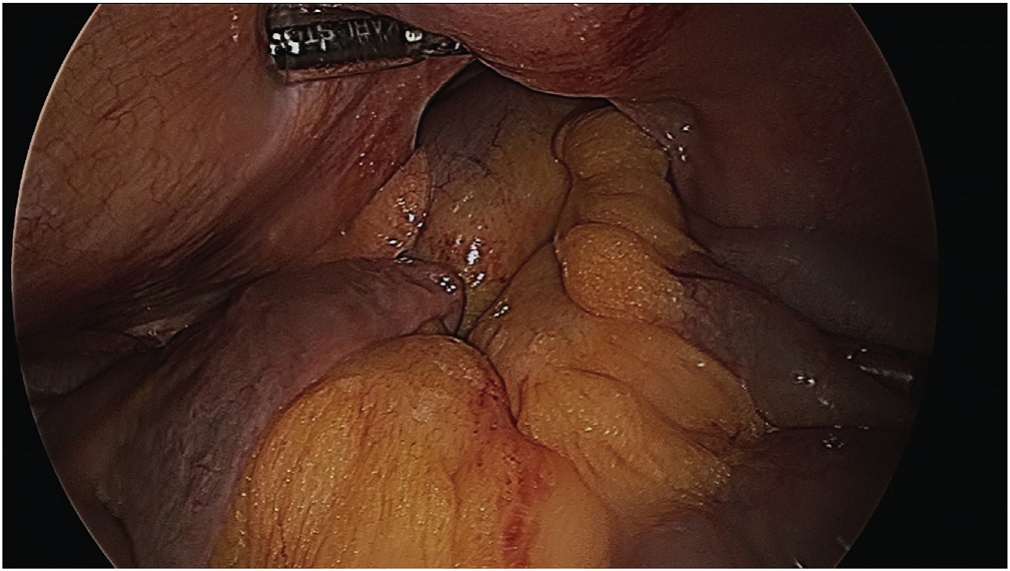
Herniation of small bowel and colon into right hemithorax.

**Fig. 3:**
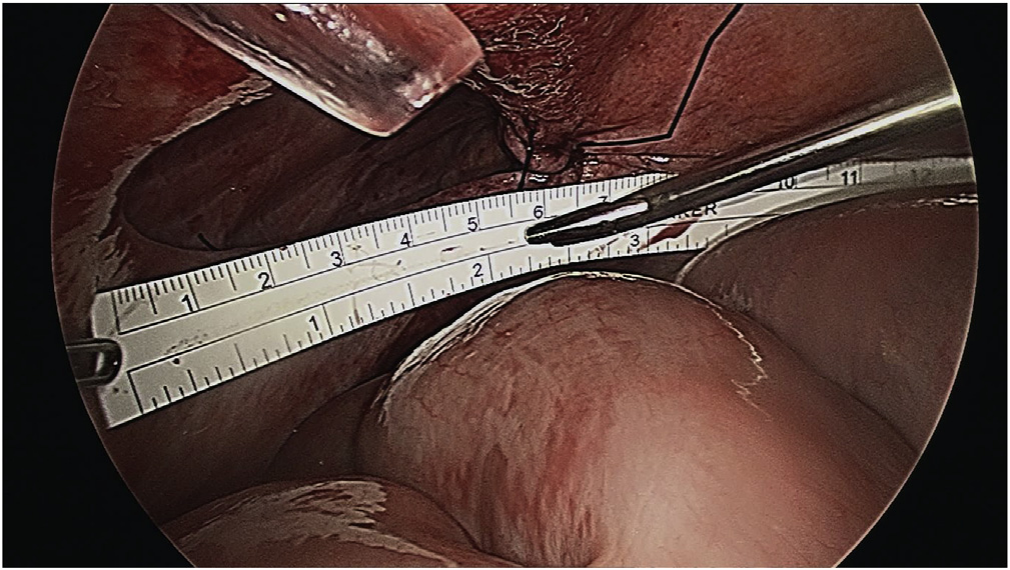
An intraoperative laparoscopic view of the right hemidiaphragm defect.

**Fig. 4:**
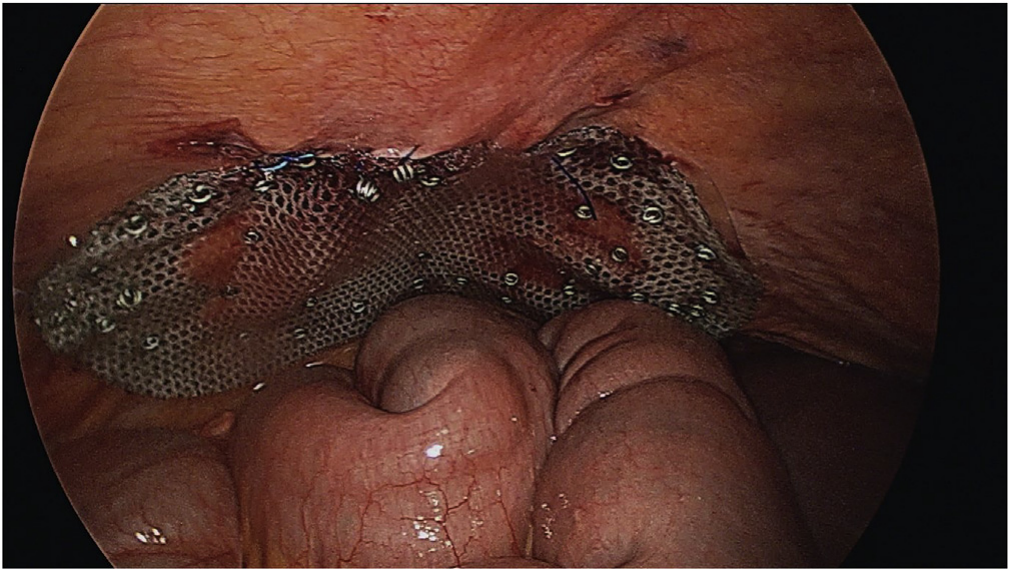
The right hemidiaphragmatic defect repaired with 100 × 150mm composite Parietex™ mesh (Medtronic, Australia), fixed with titanium tacks and non-absorbable sutures.

The patient underwent a diagnostic laparoscopy under general anaesthesia. The surgery was performed by a senior consultant oesophagogastric surgeon. A 12mm Kii Fios First Entry trochar (Applied Medical, California, USA) was performed to enter to peritoneal cavity and establish pneumoperitoneum. This was followed by insertion of three further ports under direct vision (sizes 12mm, 5mm, 5mm). Intraoperative findings confirmed a lateral right hemidiaphragmatic defect (Fig. 3), with intra-thoracic herniation of small bowel and colon (Fig. 2). The abdominal viscera were reduced into the abdominal cavity and appeared viable with no evidence of ischaemic injury. Primary closure of the diaphragmatic defect was attempted with 1 nylon, however the edges were difficult to adequately appose due to the proximity to the liver and size of the defect. Instead, the defect was bridged with a 100 × 150mm composite Parietex™ mesh (Medtronic, Australia), fixed with titanium tacks and non-absorbable sutures (Fig. 4). The patient had an uneventful postoperative recovery and was discharged home after five days. The patient remained well at a two-month follow-up visit.

## 3 Discussion

Diaphragmatic injury is a rare clinical entity which remains a diagnostic and therapeutic challenge. Clinical presentation varies from acute haemodynamic instability to delayed presentation of respiratory distress, intestinal obstruction, and/or perforation secondary to herniation of viscera into the thorax [8]. Dyspnoea remains the most common presenting symptom. Diaphragmatic injury is three times more common following a blunt abdominal trauma than penetrating trauma and often results in larger tears [4,5]. Following blunt trauma, left-sided injuries are three times more frequent than injuries to the right hemidiaphragm, attributed to a buffering effect of the liver on the right hemidiaphragm. There are two different hypotheses to explain the delay in presentation. Firstly, rupture of devitalized diaphragmatic muscle may be delayed by several days following the initial insult. The other possible explanation is that temporary plugging of diaphragmatic defect by omentum or abdominal viscera may occur, delaying the timing of symptomatic visceral herniation [1].

In 1974, Grimes described three presentation phases of diaphragmatic injury. These included (a) acute, diagnosed at the time of injury; (b) delayed, presenting after a period since the initial insult, and; (c) chronic, presenting only after intestinal obstruction or strangulation [6,9,10]. In this case, the injury manifested only after herniation of abdominal viscera caused dyspnoea and pain.

Mechanisms of injury may include lateral or frontal impact. Lateral impact delivers a shearing force on the diaphragm, whilst a frontal impact causes a rapid increase in intra-abdominal pressure. A rupture most commonly occurs at the weakest point of the diaphragm which is the posterolateral aspect between lumbar and intercostal muscle attachments. The tear extends radially and is generally longer than 100mm [11].

Chest x-ray and CT imaging are the most commonly used techniques in the diagnosis of diaphragmatic rupture. CT imaging has better sensitivity than x-ray to detect right-sided diaphragmatic injuries. It is also useful to identify concurrent thoraco-abdominal injuries before surgery. As such, it is the diagnostic imaging of choice. Diagnostic findings on CT imaging include discontinuity of the hemidiaphragm, dependent viscera sign (dependent position of the herniated viscera against the posterior ribs in the supine position), collar sign (constriction of the viscus at the site of defect), and intrathoracic herniation of abdominal contents [9,10]. In addition, Koroglu et al. demonstrated an increased diagnostic yield with the use of oral contrast [12].

The surgical management of traumatic diaphragmatic injury can be achieved via laparoscopy, thoracoscopy, laparotomy, and/or thoracotomy. The objectives of surgery include hernia reduction, defect repair, and pleural drainage [13]. The choice of surgical approach depends upon the clinical presentation, associated intrathoracic or intra-abdominal injuries, timeliness of diagnosis, and expertise of the operative surgeon. There is no consensus in the literature to direct the recommended surgical approach. According to Petrone et al. [14], transabdominal is favoured over transthoracic approach in acute traumatic diaphragmatic injury to allow for reduction of herniated viscera and to prevent missed intra-abdominal injuries. In one of the largest studies to date on this topic, Liao et al. [15] performed a comparison of open repair and laparoscopic repair which suggested that the laparoscopic approach was associated with a shorter hospital stay with no difference in recurrence or mortality. However, this was using retrospective data in a small cohort of patients (n = 24) without randomisation or blinding [15].

In contrast to a repair performed acutely, the surgical complexity and complications are greater in delayed presentation of diaphragmatic injury secondary to adhesion formation and fibrosis [15. Consequently, reports of laparoscopic repair of traumatic diaphragmatic defect, particularly those with a delayed presentation are limited. However, some authors have reported successful repair via minimally invasive techniques which avoid the morbidity of open surgery [15]. When comparing minimally invasive approaches, laparoscopy is favoured over thoracoscopy, given the requirement to use a dual-lumen endotracheal tube and single lung ventilation for thoracoscopic surgery. In the case described here, laparoscopy was performed as no significant visceral injury was identified on preoperative imaging and the operative surgeon had adequate laparoscopic expertise. Moreover, inspection of intrathoracic organs is usually possible via the defect [16].

Intra-operatively, it is important to obtain full visualization of the diaphragm. This can be done by transection of falciform ligament to visualize the right hemidiaphragm, and careful downward retraction of the greater curvature of stomach and spleen to visualize the left hemidiaphragm. After reducing all herniated intra-abdominal viscera, any devitalized diaphragmatic tissue should be debrided [14].

A small diaphragmatic defect may be repaired with non-absorbable suture material or, rarely, with absorbable sutures. A prosthetic mesh repair is recommended for larger defects to minimise tension, which is especially useful in delayed repair due to adhesion formation and diaphragmatic atrophy [13]. There is no available evidence currently regarding mesh selection, rates of recurrence and/or infection following mesh repair. Further research is needed in this field to identify the optimum surgical technique for diaphragmatic injury.

## 4 Learning points

• Diaphragmatic injury is missed on initial assessment in 25% of cases. Right-sided hemidiaphragmatic injury is less common and is often a missed diagnosis.
• A missed diagnosis following acute injury can later result in life-threatening complications including cardiorespiratory compromise and bowel strangulation secondary to the herniation of intra-abdominal contents into the hemithorax.
• Computed tomography (CT) of the Chest and Abdomen remains the diagnostic modality of choice for traumatic diaphragm injury.
• Laparoscopic repair of delayed diaphragmatic defect with mesh is achievable and avoids the morbidity associated with open surgery.

## Ethical approval

Not applicable.

## Sources of funding

None.

## Author contribution

Study concepts and study design: Bee Shan Ong, Paul Heitmann, Jon Shenfine.

Data acquisition: Bee Shan Ong.

Data analysis and interpretation: Bee Shan Ong.

Manuscript preparation: Bee Shan Ong.

Manuscript editing: Bee Shan Ong, Paul Heitmann.

Manuscript review: Jon Shenfine.

## Research registration number

NOT applicable.

1. Name of the registry:

2. Unique Identifying number or registration ID:

3. Hyperlink to your specific registration (must be publicly accessible and will be checked).

## Guarantor

Bee Shan Ong.

## Consent

Written informed consent was obtained from the patient for publication of this case report and accompanying images. A copy of the written consent is available for review by the Editor-in-Chief of this journal on request.

## Provenance and peer review

Not commissioned, externally peer reviewed.

## Declaration of competing interest

None.
